# Physicians’ Knowledge, Practices, and Perceptions of Reporting Communicable Diseases at Primary Health Care Centers in Jeddah, Saudi Arabia: A Cross-Sectional Study

**DOI:** 10.7759/cureus.32558

**Published:** 2022-12-15

**Authors:** Mohammed H Alshehri, ‏Amal H Alghamdi, Abeer A Subke, Sultan A Alamri, Hanan H Al Muwallad, Sultan A Alghamdi, Ibrahim M Asiri, Noura N Alotaibi, Osama A Bugis

**Affiliations:** 1 Public Health Administration, Ministry of Health, Jeddah, SAU; 2 Public Health Administration, Saudi Board of Preventive Medicine Program, Ministry of Health, Jeddah, SAU; 3 Family Medicine, King Abdulaziz Hospital, Ministry of Health, Jeddah, SAU; 4 Family Medicine, King Abdullah Medical Complex, Ministry of Health, Jeddah, SAU; 5 Laboratory - Clinical Immunology, King Fahad General Hospital, Ministry of Health, Jeddah, SAU

**Keywords:** primary health care centers, disease surveillance, disease reporting, communicable disease, jeddah, saudi arabia

## Abstract

Background

Jeddah has the highest international traffic and is among the most diverse cities in Saudi Arabia. The chance of importing an emerging communicable disease is significant, particularly during the religious seasons. Therefore, timely and accurate reporting of communicable diseases at primary health care centers (PHCCs) is crucial.

Objectives

The main objective of this study was to assess physicians’ knowledge, practices, and perceptions of reporting communicable diseases at PHCCs in Jeddah, Saudi Arabia.

Methods

The study was a cross-sectional study comprising 143 physicians from all PHCCs in Jeddah from October 2017 to February 2018. An electronic questionnaire was used to collect data to assess the physicians’ knowledge, practices, and perceptions related to reporting communicable diseases at PHCCs.

Results

A total of 106 physicians participated in the study. Although only 21.7% of the physicians had received training on reporting communicable diseases, the average knowledge score for the six commonly reported diseases was 72%. More than half (58.5%) of the physicians indicated that they had reported at least one disease. However, there was no agreement on who should be responsible for reporting communicable diseases at PHCCs. Furthermore, some obstacles were perceived that could prevent disease reporting, including physicians not knowing which diseases to report (66%), not knowing how or whom to report to (54.7%), and a limited diagnostic or laboratory capacity (52.8%).

Conclusions

Reporting communicable diseases at PHCCs was of adequate quality. However, some obstacles must be addressed, and regular applied training must be provided. More extensive assessments are needed to improve the reporting of communicable diseases locally and nationally.

## Introduction

Communicable diseases continuously rank among the highest causes of mortality worldwide [1]. Consequently, numerous countries have implemented surveillance frameworks capable of systematically collecting, updating, analyzing, and interpreting vital information on an ongoing basis for effective control and management of communicable diseases [2]. Therefore, communicable disease surveillance systems (CDSSs) are essential in providing countries with the required information to monitor and act upon communicable diseases [3].

The Saudi Ministry of Health (MOH) adopted the primary health care (PHC) strategy as a fundamental approach to comprehensive preventive health services. One of the vital aspects of this approach is the prevention and control of communicable diseases [4]. The MOH provides primary care services through a comprehensive network of primary health care centers (PHCCs) integrated with hospitals and specialized medical centers [5]. Within this structural organization, since 2012, the MOH has implemented an online-based system, the Health Electronic Surveillance Network, enabling a smooth and timely flow of communicable disease information between facilities [5,6].

In most countries, including Saudi Arabia, CDSSs depend highly on physicians’ obligatory reporting of cases. Primary care physicians are expected to act as the first and most qualified reporting sources for the country’s central information system [7]. Despite the need and responsibility to report communicable diseases, incomplete and inaccurate reporting by physicians diminishes the usefulness of the reporting system [8,9]. Physicians’ lack of knowledge of how and when they are required to report has frequently been described as affecting their reporting of diseases [10].

Jeddah remains vulnerable to health threats with a moving multicultural population of four million and yearly international traffic of over 21 million [11,12]. Accordingly, there is a high chance of importing an emerging communicable disease, especially with the increasing international traffic during the religious seasons of Umrah and Hajj [13].

Improving the capacity of PHCCs to detect outbreaks early and rapidly respond before they become a public health concern is tied to timely and accurate reporting of communicable diseases at the level of the PHC [8,14,15]. Therefore, in this study, we assessed physicians’ knowledge, practices, and perceptions of reporting communicable diseases at PHCCs in Jeddah, Saudi Arabia.

## Materials and methods

Study design, setting, and population

This study was a cross-sectional study comprising all PHCCs in Jeddah (n=45) from October 2017 and February 2018. From each PHCC, all available full-time physicians with at least one year of work experience in PHC were selected.

Sampling and sample size

After excluding the pilot study’s PHCCs (n=3), all of the remaining PHCCs (n=42) were included in this study. From each PHCC, a list of registered physicians was obtained, totaling 304 physicians. Based on the selection criteria, a total of 147 physicians were included.

Data collection tool

An electronic questionnaire was used to collect data about the knowledge, practices, and perceptions of reporting diseases required by CDSS among physicians at PHCCs. It was developed by Lafond et al. [15] in collaboration with epidemiologists from the Nigerian Federal Ministry of Health and the US Centers for Disease Control and Prevention. The questionnaire elements included physicians’ socio-demographic information, knowledge and practices of reporting communicable diseases, and perceptions about reporting responsibility and obstacles to disease reporting.

Data analysis

Stata Statistical Software (StataCorp, College Station, USA.) was used to analyze collected data. Categorical variables were computed numerically as frequencies and percentages of each category.

Pilot study

The pilot study randomly included three out of the 45 PHCCs (n=3) to test the questionnaire’s feasibility, validity, and reliability. The questionnaire was intentionally adapted for the PHCCs in Jeddah with acceptable reliability (Cronbach’s α = 0.73).

Ethical considerations

Jeddah Health Affairs Research Ethics Committee (H-02-J-002) issued ethical approval number A00505 to conduct this study. Informed consent was obtained from all participants. Collected data were de-identified and not disclosed except for the study’s purpose.

## Results

Out of 143 selected physicians, 127 consented to participate; however, 21 did not complete the questionnaire. The participating physicians were 106, with a response rate of 74.1%.

Socio-demographic information

Table 1 shows that more than half of the physicians (58.5%) were females. The ages ranged from 25 to 51 years, with a median age of 32. The inter-quartile range of years of experience in PHC was four to eight years, with a median of 5.5 years. About half of the physicians (47.2%) specialized in family medicine. The other half (52.8%) did not hold any specialty after their medical college education. Only 21.7% had received training on CDSS, of which 68% was within the last two years.

**Table 1:** Socio-demographic characteristics of the physicians PHC: Primary Health Care; CDSS: Communicable Disease Surveillance System

Characteristics	Total (n=106)
Gender	
Male	44 (41.5%)
Female	62 (58.5%)
Age (years)	
26–30	24 (22.6%)
31–35	55 (51.9%)
36–40	19 (17.9%)
>40	8 (7.6%)
Experience in PHC (years)	
1–5	53 (50%)
6–10	36 (34%)
11–15	15 (14.1%)
>15	2 (1.9%)
Level of Medical Education	
General practice	56 (52.8%)
Family medicine specialty	50 (47.2%)
Community medicine specialty	0 (0%)
Training	
Received training on CDSS	23 (21.7%)

Disease reporting

In response to questions about disease reporting, all physicians knew that poliomyelitis requires immediate reporting. However, only 38% of physicians knew that tuberculosis requires routine reporting. Physicians’ correct responses ranged from 61% to 86% when asked whether diseases like measles, dengue fever, chickenpox, and hepatitis B require immediate or routine reporting. The average knowledge score for these six commonly reported diseases was 72%. When participants were asked who should be responsible for reporting communicable diseases, there were various responses (Table 2). Preventive medicine teams (50%) and general managers or medical directors (34.9%) were most frequently mentioned, but health inspectors (26.4%), physicians (23.6%), laboratory technicians (5.7%), and nurses (2.8%) were also listed.

**Table 2:** Physicians’ perception of who is responsible for reporting communicable diseases

Who is responsible for reporting	Total (n=106)
General manager or medical director	37 (34.9%)
Immediate supervisor/consultant	0 (0%)
Communicable disease consultant	12 (11.3%)
Preventive medicine team	53 (50%)
Medical record officer	0 (0%)
A designated officer	19 (17.9%)
Physicians	25 (23.6%)
Nurses	3 (2.8%)
Laboratory technicians	6 (5.7%)
Health inspectors	28 (26.4%)

Regarding physicians’ own experience in reporting diseases, more than half (58.5%) of them indicated that they had reported at least one disease. Chickenpox, measles, and dengue fever were the most commonly reported diseases.

Reporting obstacles

Table 3 provides the physicians’ perceived obstacles to reporting communicable diseases. Leading responses of obstacles relating to knowledge and attitudes comprised not knowing which diseases to report (66%), not knowing how or whom to report to (54.7%), and not knowing that they should report (51.9%). Additionally, time and resource concerns included limited diagnostic or laboratory capacity (52.8%) and physicians being too busy to report (43.4%). Further obstacles included the lack of infrastructure or reporting system (27.4%) and the belief that reporting would not activate any higher-level response (15.1%).

**Table 3:** Physicians’ perceived obstacles to reporting communicable diseases

Obstacles to Reporting	Total (n=106)
Knowledge and Attitudes	
Not knowing they should report	55 (51.9%)
Not feeling it is important	16 (15.1%)
Not knowing how or whom to report to	58 (54.7%)
Not knowing which diseases to report	70 (66%)
Not believing it is their position to report	28 (26.4%)
Never have seen a disease required to report	22 (20.8%)
May want to protect patient confidentiality	0 (0%)
Time and Resources	
Limited diagnostic or laboratory capacity	56 (52.8%)
Too busy to report	46 (43.4%)
Too complicated or cumbersome reporting process	43 (40.6%)
Lack of the appropriate materials (e.g., forms, internet)	44 (41.5%)
System Infrastructure	
Lack of infrastructure or reporting system	29 (27.4%)
Believing higher-level management will take no action	16 (15.1%)

Post-reporting actions

Table 4 presents a comparison of higher-level actions expected by physicians who had never reported communicable diseases (n=44) with those observed by physicians who had reported at least one communicable disease (n=62). Actions such as giving advice on disease management and treatment and providing referral and transportation services exceeded expectations. However, most of the observed actions were less than what was expected. For example, the observed action of providing public education was 150.6% lower than expected, followed by alerting the community, which was 95.7% lower.

**Table 4:** Expected versus observed higher-level actions after reporting a communicable disease Expected: Higher-level actions were expected by physicians who never had reported communicable diseases. Observed: Higher-level actions were observed by physicians who had reported at least one communicable disease.

Higher-Level Actions	Expected (n=44)	Observed (n=62)	% Difference
Verify/confirm case and/or conduct investigation	41 (93.2%)	48 (77.4%)	-18.5%
Provide medical supplies for patient treatment	11 (25%)	13 (21%)	-17.4%
Assist with controlling the spread of disease	22 (50%)	27 (43.5%)	-13.9%
Alert the other government entities	8 (18.2%)	6 (9.7%)	-60.9%
Alert district, city, and community	6 (13.6%)	3 (4.8%)	-95.7%
Give advice on disease management and treatment	12 (27.3%)	32 (51.6%)	61.6%
Provide public enlightenment/education	15 (34.1%)	3 (4.8%)	-150.6%
Provide referral and transport services	1 (2.3%)	16 (25.8%)	167.3%

## Discussion

Jeddah has the highest international traffic in Saudi Arabia, connecting millions of people from over 180 countries to Makkah for religious pilgrimages, which turns it into one of the most diverse mass gatherings globally [16]. The myriad challenges from such mass gatherings give the Saudi MOH the privilege of experience and the capacity to handle communicable diseases. However, the chance of importing communicable diseases and possibly spreading them nationally and globally is still there [17]. Therefore, although the CDSS in Saudi Arabia is well-functioning and enhanced, improvements in CDSS capabilities, especially the quality of disease reporting, pose a formidable challenge for every country, as policymakers require an efficient and precise reporting system at local and national levels [18].

In this study, we assessed the physicians’ reporting of communicable diseases in Jeddah at the level of PHC, representing the primary level of the health system that provides health care services to the public sector.

Regarding physicians’ knowledge of communicable diseases, the average score for the six commonly reported diseases was 72%, which is better than the results from the previous study conducted in Jeddah in 2009 [14]. The variation of scores between diseases may be attributed to insufficient training, where only 22% of physicians reported receiving training on CDSS recently. The physicians also reported a lack of accessible learning materials and the effect of increased concern for emerging diseases over other diseases. However, the low score of knowledge is of concern and needs improvement.

The study also noted no agreement between physicians on when and to whom they should report communicable diseases. The leading obstacles to reporting included not knowing which diseases to report, not knowing how or whom to report to within PHCC, and limitations in PHC laboratories’ diagnostic capacity. In addition, actions from higher levels observed by physicians fell below expectations, which could be attributed to inadequate training or limited feedback from those higher levels.

Unfortunately, such issues with disease reporting exist all across the world. For example, a study from Nigeria [19] involving health care professionals showed that almost 25% of the health care workers who were aware of disease reporting forms did not know which form to use for reporting purposes. Moreover, 74% had never reported disease or received specific training in disease reporting. Another study from Nigeria [15] focused on physicians’ observed obstacles to reporting. Findings showed that appropriate instructions were not being received from the superior authorities for disease-reporting purposes. Finally, a systematic review article by Janati et al. [18] concluded that disease-reporting systems all across the world have problems of a similar nature.

Limitations of the study

This study focused only on the PHC level among several other levels in the MOH health care system. Additionally, sectors other than MOH, such as the military and private sectors, were not studied; however, they have various structures and functions, which may affect their findings.

## Conclusions

Based on the findings, the reporting of communicable diseases at the level of PHC in Jeddah was of adequate quality compared with other countries. The majority of physicians had the required knowledge of communicable diseases and had reported at least once, with various obstacles to disease reporting that need effective solutions.

To improve the reporting of communicable diseases at PHCCs in Jeddah, we recommend providing applied training on CDSS for PHC providers, especially physicians, which should be mandatory and planned as an annual activity. Details of trained providers should be maintained in a database and updated regularly. Training materials need to be clear, sufficient, and available on demand. Furthermore, higher-level feedback is crucial to satisfy the communication loop and motivate PHCCs to improve their disease reporting.

More extensive assessments are needed to improve the reporting of communicable diseases locally and nationally.
